# Causative agent for perioperative anaphylaxis in a child with autism successfully identified using the intradermal test under general anesthesia

**DOI:** 10.1186/s40981-024-00733-0

**Published:** 2024-08-08

**Authors:** Yasuhiro Amano, Kumi Mizutani, Yuki Kato, Tasuku Fujii, Akiko Yagami, Takahiro Tamura

**Affiliations:** 1 Department of Anesthesiology, Nagoya University Graduate School of Medicine, 65 Tsurumai-Cho, Showa-Ku, Nagoya, 466-8550 Japan; 2 Department of Pediatrics, Fujita Health University School of Medicine, Aichi, Japan; 3 Fujita Health University General Allergy Center, Bantane Hospital, Nagoya, Japan; 4 Department of Allergology, Fujita Health University School of Medicine, Aichi, Japan

**Keywords:** Anaphylaxis, Intradermal test, General anesthesia, Autism

## Abstract

**Background:**

The skin-prick and intradermal tests are the main diagnostic methods used to identify the causative agent in patients with suspected perioperative anaphylaxis. Although the intradermal test is more sensitive than the skin-prick test, multiple intradermal injections can be painful for children. Here, we present the case of a child with autism and suspected perioperative anaphylaxis. The causative agent was successfully identified using the intradermal test under general anesthesia.

**Case presentation:**

An 8-year-old boy with autism developed anaphylaxis during general anesthesia for the fourth cleft lip and palate surgery. An allergic workout was performed, but both the skin-prick and basophil activation tests for suspected causative agents yielded negative results. The patient was afraid of multiple injections, and an intradermal test was performed under general anesthesia by anesthesiologists and allergists. Piperacillin was confirmed as the causative agent, and subsequent surgery using the same anesthetic agents without piperacillin was uneventful.

**Conclusions:**

Concerted efforts should be made to identify the causative agent for diagnosing perioperative anaphylaxis.

## Background

Diagnosing the causes of perioperative anaphylaxis is challenging. A systematic diagnostic approach is required, and differential diagnoses should be made. Although intradermal skin tests (IDT) are useful for determining the causative agent, they can be painful for children and are not often performed. However, the causative agent is often hard to determine without an IDT, and future anesthesia safety cannot be ensured. Collaboration between anesthesiologists and allergists allows IDT to be performed under appropriate sedation in children. Here, we describe a case of successful identification of the culprit drug for perioperative anaphylaxis using IDT under general anesthesia in a child with autism. This case was also included in an observational study of perioperative anaphylaxis approved by the institutional review board of Nagoya University Hospital (approval number: 2020–0020). Written informed consent for publication was obtained from the patient’s parents.

## Case presentation

An 8-year-old, 137-cm, 44-kg boy; + 2 standard deviations compared to average 8-year-old Japanese boys, diagnosed with autism was scheduled for the fourth cleft lip and palate surgery under general anesthesia at a dental hospital. The three general anesthesia procedures performed 8, 7, and 3 years prior had been uneventful. The third of those procedures was induced using sevoflurane, rocuronium, and fentanyl. Betamethasone was administered to prevent postoperative swelling, and piperacillin was administered as a prophylactic antibiotic. The fourth general anesthesia was induced using sevoflurane, propofol, rocuronium, fentanyl, and remifentanil, and betamethasone and piperacillin were again administered, as in the third cleft lip and palate surgery. Endotracheal intubation was uneventful; however, the patient developed elevated airway pressure 18 min after tracheal intubation, followed by systemic rash and hypotension with a systolic blood pressure of 64 mmHg. The patient was unable to ventilate by volume control ventilation at a maximum airway pressure of 30 cmH_2_O, and the anesthesiologists ventilated him manually with a maximum airway pressure of 70 cmH_2_O. Because perioperative anaphylaxis was suspected, the procedure was discontinued, and the patient was administered 0.2 mg of intramuscular adrenaline. Serum tryptase was elevated to 7.6 μg/L two hours after symptom onset compared to the level measured 19 h after the onset; 3.0 μg/L.

Four months after the event, the patient presented to the Department of Pediatrics of Fujita Health University Bantane Hospital, where an allergic workout was performed (approval number: HM23-458). Because the patient was afraid of multiple injections, we first performed a basophil activation test (BAT) for all drugs administered before the event and measured latex-specific IgE. Both tests yielded negative results. Next, we performed a skin prick test (SPT); however, the results were negative. In patients with symptoms in multiple organs, anaphylaxis cannot be excluded based on negative SPT and BAT results. Therefore, IDT was planned to identify the culprit drug, however, performing IDT was challenging. Although the patient tolerated the SPT, we assumed that multiple intradermal injections during IDT would be intolerable and potentially traumatic. Hence, we decided to perform IDT under general anesthesia in the operating room at Nagoya University Hospital.

**Fig. 1:**
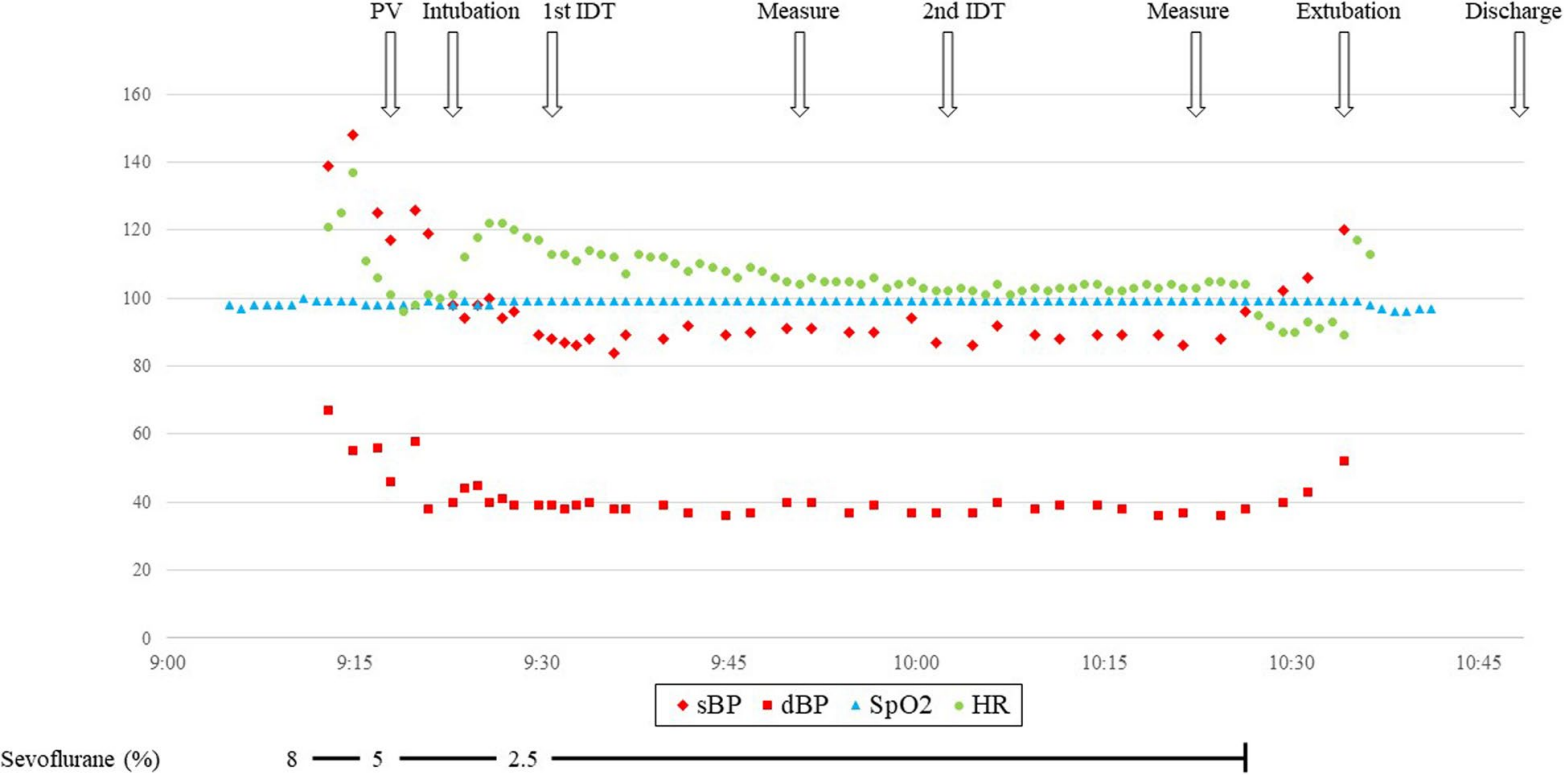
Anesthetic chart for the intradermal tests. dBP, diastolic blood pressure; HR, heart rate; IDT, intradermal test; PV, peripheral venous catheter insertion; sBP, systolic blood pressure

Figure 1 shows the anesthetic chart for the IDT. General anesthesia was induced using sevoflurane and oxygen, and a peripheral venous catheter was inserted after topical disinfection using alcohol wipes. A supraglottic airway device (i-gel) was inserted uneventfully, and no skin symptoms, wheezing, or elevated airway pressure were observed. Spontaneous breathing was maintained throughout the test. All drugs other than piperacillin were diluted as described in previous guidelines [1, 2]. The maximum non-irritative concentration for piperacillin was set at 10 mg/mL. After confirming that the patient’s hemodynamics were stable, we initiated IDT. Saline and histamine were used as the negative and positive controls, respectively. The 1st IDT was performed using a tenfold dilution of the suggested maximum non-irritative concentration for IDT. The injection volume was 0.02 mL for all drugs, and a 3–5 mm wheal was created on the patient’s forearm. Wheal diameter was measured using a digital caliper at baseline (Wi) and 20 min after injection (W20). Test positivity was determined based on the following criterion: W20 ≥ Wi + 3 mm with surrounding flare [1].

**Fig. 2:**
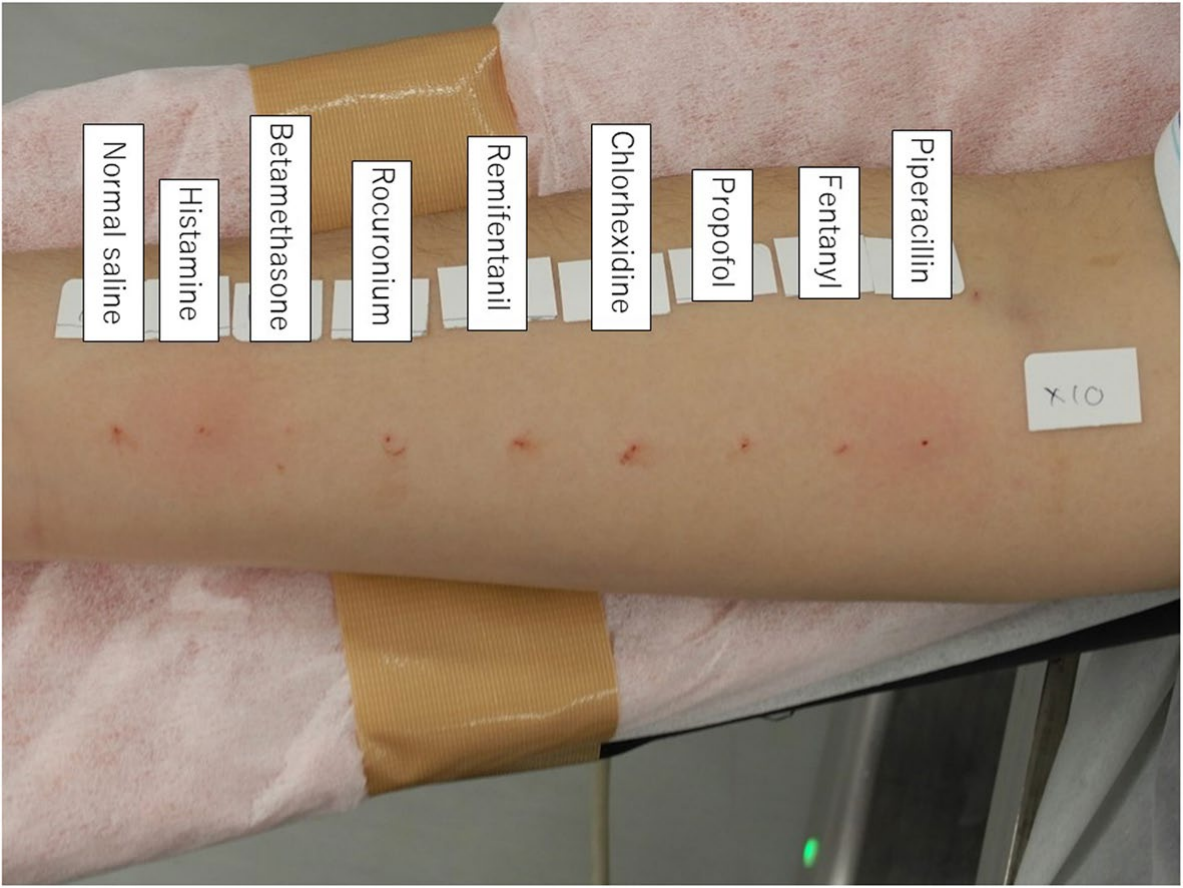
Results of the 1st intradermal test procedure

**Fig. 3:**
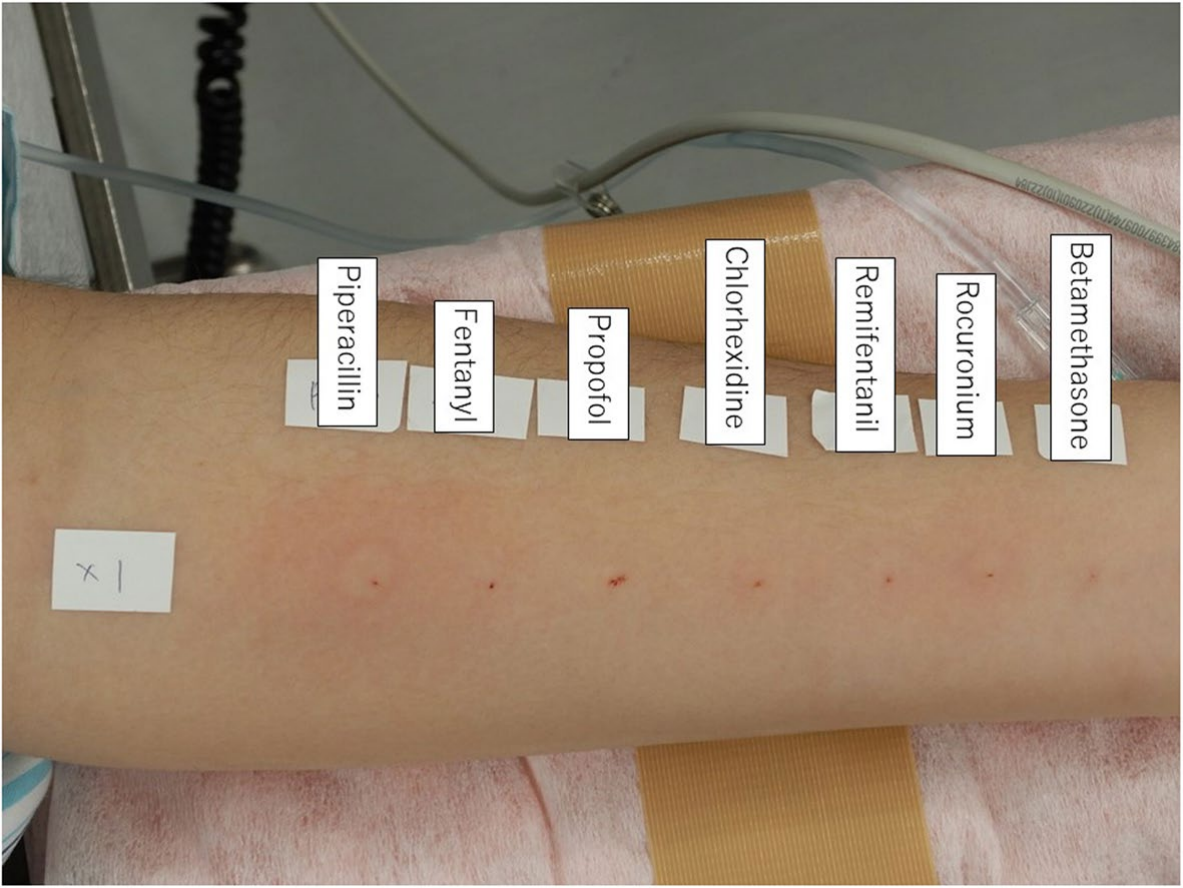
Results of the 2nd intradermal test procedure

Piperacillin (1 mg/mL) showed positive results (Fig. 2, Table 1). Considering the possibility of false negatives other than piperacillin, we performed a second IDT for all drugs at the suggested maximum non-irritative concentration. Piperacillin (10 mg/mL) again yielded positive results, whereas the other drugs yielded negative results (Fig. 3 and Table 1). The patient recovered from general anesthesia uneventfully, and we concluded that piperacillin was the causative agent. Subsequent surgery, using the same anesthetics and fosfomycin as the prophylactic antibiotic, was uneventful.

**Table 1:** Results of the intradermal tests

	**1st procedure**					**2nd procedure**				
**Drug**	**Concentration (mg/mL)**	**Wi (mm)**	**W20 (mm)**	**Flare (mm)**	**Judgement**	**Concentration (mg/mL)**	**Wi (mm)**	**W20 (mm)**	**Flare (mm)**	**Judgement**
Saline	9	4.6	0	0	-	NA	NA	NA	NA	NA
Histamine	10	3.8	9.2	28	+	NA	NA	NA	NA	NA
Betamethasone	0.04	3.2	0	0	-	0.4	4.3	0	0	-
Rocuronium	0.005	3.3	0	0	-	0.05	3.5	5.6	22	-
Remifentanil	0.0005	3.8	0	0	-	0.005	3.5	0	0	-
Chlorhexidine	0.0002	3.5	0	0	-	0.002	4.5	4.7	0	-
Propofol	0.1	4.3	0	0	-	1	3.8	0	0	-
Fentanyl	0.0005	3.7	0	0	-	0.005	3.1	0	0	-
Piperacillin	1	3.4	7.6	30	+	10	3.9	8.6	44	+

IDT, intradermal test; NA, not applicable; Wi, initial diameter of the wheal immediately after injection; W20, diameter of the wheal 20 min after injection

## Discussion

Skin tests, such as SPT and IDT, are universally used to investigate the causative agents of perioperative anaphylaxis [1]. IDT is more sensitive but painful compared to SPT and is often poorly tolerated by small children [3]. In our case, performing IDT in the usual manner was challenging because the patient was afraid of needles and multiple injections. One possible approach was to avoid all suspected drugs and reattempt general anesthesia without determining the causative agent because of the inability to perform IDT. However, the list of suspected drugs included many drugs essential for general anesthesia with few alternatives, which can limit the anesthesiologists’ options; therefore, this approach was undesirable [4]. Moreover, because symptoms occurred 18 min after tracheal intubation, the timing of onset was considered late for anaphylaxis [5], even if it was caused by the drugs administered during induction. However, the timing of onset and administration of suspected drugs should not be used to predict the culprit [6]. Moreover, the patient presented with multi-organ symptoms and showed elevated serum tryptase levels [7, 8], and anaphylaxis was difficult to rule out. Recently, the Hypersensitivity Clinical Scoring Scheme has been developed as an objective tool for assessing the likelihood of anaphylaxis [5]. Our patient’s score was 22, pointing to a high probability of anaphylaxis. After discussing the risks and benefits of IDT with the allergists and anesthesiologists, we decided to perform an IDT under sedation.

A few studies on skin tests in children have been reported [3], and efficacy and safety of sedation during skin testing in children have not been established. To the best of our knowledge, only one case of IDT performed under sedation in a 2-year-old girl has been reported [9]. In that case, general anesthesia was induced using intramuscular ketamine and maintained using continuous sevoflurane inhalation. Since giving the patient a muscle injection or securing an intravenous line when awake was challenging, we performed slow induction with inhalation of sevoflurane. Moreover, sevoflurane can be used alone for the maintenance of anesthesia; therefore, we assumed that it would be appropriate for sedation for IDT if the airway was secured using a supraglottic device. Although sevoflurane was one of the suspected causative drugs for anaphylaxis, few cases of sevoflurane anaphylaxis have been reported despite its widespread use [10, 11]. We estimated that the risk of anaphylaxis recurrence due to sevoflurane was extremely low. Rather, we believed that re-administering sevoflurane would be more beneficial and would confirm its safety for future anesthesia. IDT under general anesthesia using sevoflurane alone was uneventful, and we believe that our decision was correct.

The methodology and interpretation of IDT should be standardized [12]. We performed the IDT based on the European Network for Drug Allergy Working Group method [1]. We set the maximum non-irritating drug concentration for suspected causative drugs based on two recommendations [1, 2]. Because piperacillin dilutions were not included, we set the maximum non-irritating piperacillin concentration at 10 mg/mL. A previous study reported that IDTs performed on healthy individuals using 1 mg/mL and 20 mg/ml piperacillin yielded negative results [13]. Although it is likely that the previous study comprised of adults only, we assumed that those piperacillin concentrations could apply to our patient because the general recommendations of adults’ skin tests have previously been applied to children as well [3]. Since the piperacillin concentrations used in both IDTs in our patient were < 20 mg/mL, our results were unlikely to be false positives. Other in vitro tests could have complemented the positive result for piperacillin in the IDT; however, BAT results were negative, and a reagent measuring piperacillin-specific IgE is not commercially available in Japan. Some drugs, such as neuromuscular blocking agents and opioids, often yield false-positive results in skin tests [14]. In fact, the wheal was enlarged and surrounded by flare for rocuronium at a concentration of 50 μg/mL (Fig. 3, Table 1). We used a digital caliper to accurately measure the wheal for rocuronium, and it did not meet the positivity criteria for IDT. Although general anesthetics can cause immunosuppression [15], we believe that they did not affect the skin tests results because the histamine used for the positive control yielded a positive reaction. The subsequent surgery was completed using same anesthetic drugs without piperacillin, and piperacillin anaphylaxis was suggested. We investigated previous anesthetic records, and piperacillin was administered prophylactically during the third surgery under general anesthesia performed three years prior to the anaphylaxis. We assumed that the patient was sensitized to piperacillin during the third surgery under general anesthesia and developed anaphylaxis during the fourth surgery under general anesthesia.

In conclusion, concerted efforts should be made to perform IDT and successfully identify the causative agent. Collaboration between anesthesiologists and allergists can lead to a definitive diagnosis of perioperative anaphylaxis.

## Data Availability

Data relevant to this case report are not available for public access because of patient privacy concerns but are available from the corresponding author on reasonable request.

## Abbreviations

IDT: Intradermal skin tests
BAT: Basophil activation test
SPT: Skin prick test
